# Identification of genetic risk loci for depression and migraine comorbidity in Han Chinese residing in Taiwan

**DOI:** 10.3389/fpsyt.2022.1067503

**Published:** 2023-01-10

**Authors:** Ming-Chen Tsai, Chia-Lin Tsai, Chih-Sung Liang, Yu-Kai Lin, Guan-Yu Lin, Chia-Kuang Tsai, Po-Kuan Yeh, Yi Liu, Kuo-Sheng Hung, Fu-Chi Yang

**Affiliations:** ^1^Department of Neurology, Tri-Service General Hospital, National Defense Medical Center, Taipei City, Taiwan; ^2^Department of Psychiatry, Beitou Branch, Tri-Service General Hospital, National Defense Medical Center, Taipei City, Taiwan; ^3^Department of Neurology, Songshan Branch, Tri-Service General Hospital, National Defense Medical Center, Taipei City, Taiwan; ^4^Center for Precision Medicine and Genomics, Tri-Service General Hospital, National Defense Medical Center, Taipei City, Taiwan

**Keywords:** migraine, depression, single nucleotide polymorphism (SNP), genetic variant, Han Chinese, susceptibility loci

## Abstract

**Introduction:**

The genetic association between depression and migraine has not been well investigated in Asian populations. Furthermore, the genetic basis of depression and comorbid migraine subtypes remains nebulous. Hence, in the current study we investigate the susceptibility loci associated with depression and migraine comorbidity in the Han Chinese population in Taiwan.

**Methods:**

We perform a genome-wide association study involving 966 migraine patients, with or without comorbid depression. Genotyping is performed using participant genomic DNA. Association analyses are performed for the entire migraine cohort (subgroups: episodic migraine, chronic migraine, and migraine with or without aura).

**Results:**

Results show that the single nucleotide polymorphism variants of the *CDH4* intron region (rs78063755), *NTRK3-AS1* downstream region (rs57729223), and between *LINC01918* and *GPR45* (rs2679891) are suggestively associated with depression. Twenty additional susceptibility loci occur within the subgroups. A multivariate association study demonstrated that a variant in the intron region of *CDH4* rs78063755 was associated with Beck Depression Inventory and Migraine Disability Assessment scores.

**Discussion:**

The findings of this study identify several genetic loci suggestively associated with depression among migraine patients in the Han Chinese population. Moreover, a potential genetic basis has been characterized for depression and migraine comorbidity, thus providing genetic candidates for further investigation.

## 1. Introduction

Depression is one of the most common neuropsychiatric and disabling disorders with an approximate 10% prevalence worldwide (1). The etiology of depression is multifactorial with a complicated neurobiological and psychosocial framework (2). Meanwhile, migraine is a common neurological disorder characterized by chronic and recurrent headache events predominantly in females, affecting 10–20% of adults worldwide (3). Analysis of the 2019 Global Burden of Disease data revealed that migraine was the second most disabling disease worldwide and the first among young women (4). Of note, migraine comorbid with depression has been observed in clinical studies (5, 6) far more commonly than expected by chance. In fact, the two diseases have a reported bidirectional association; that is, migraine increases the risk of major depression, and major depression increases the risk of migraine (7). Indeed, in migraine patients with depression, the clinical symptoms may be exacerbated and are more difficult to treat (8).

Previous research has demonstrated that epidemiological co-occurrence of diseases may not be random and can indicate possible shared factors, including genetic, environmental, or interactions between the two (9). Meanwhile, large health data banks with detailed medical information provides a significant opportunity for the exploration of the etiologies of complex disease comorbidities. A UK Biobank cohort study with 117,392 subjects supported the relationship between migraine and depression comorbidity (10). In particular, characterization of a shared genetic background may direct further personalized diagnosis and treatment. Indeed, the genetic association of migraine and depression comorbidity is well-documented in genome-wide association studies (GWASs) within European populations (11–13). Genetic risk score analysis conducted by Ligthart et al. (11) indicated that migraines with or without depression comorbidity are genetically distinct disorders and the subgroup of patients with depression and migraine comorbidity were genetically much similar to patients with depression. A genetic association between migraine and depression comorbidity was also observed in the Netherlands Twin Registry. By investigating discordant monozygotic twins, the study found that only the twin that had depression had increased risk for migraine (12). Since most studies addressing this issue were conducted in non-Asian populations, whether these GWASs finding are applicable in an Asian population remains questionable. Considering that migraine is a complicated neurovascular disease with a strong genetic component that differs for each subtype (14), we hypothesize that migraine patients with different subtypes may develop depression *via* different mechanisms.

To the best of our knowledge, no previous study has reported on the genetic basis of overall and subtype migraine, nor on depression comorbidity in an Asian population. Therefore, in this study, we perform a GWAS on a migraine cohort in a tertiary hospital in Taiwan, to identify genetic loci associated with comorbid depression and migraine. Furthermore, we divide the migraine cohort based on migraine subtypes: episodic migraine, chronic migraine, migraine with aura, and migraine without aura. We then analyze possible susceptible genetic loci by comparing the genotype of various migraine subtypes with and without depression.

## 2. Materials and methods

### 2.1. Participants

**FIGURE 1:**
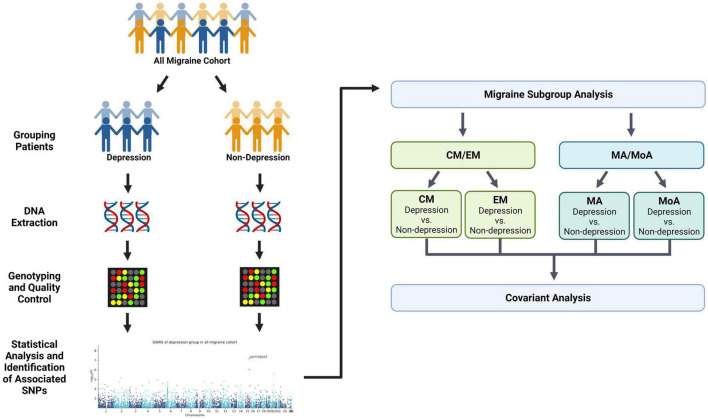
Flowchart showing the pipeline of the covariant analysis in depression and migraine. We divided migraine patients into two groups (non-depression and depression) and performed the first phenotype association study with depression (Table 2). Next, samples were stratified into four migraine subtypes: episodic migraine (EM), chronic migraine (CM), aura (MA), and non-aura (MoA). Each group was further divided into two subgroups based on the presence and absence of comorbid depression. The results of the quantitative trait study in the second stratification are shown in Table 3. Finally, we compared the variants in the migraine group and subgroups to summarize the covariant in the study. This figure was created with Biorender.com.

A total of 966 patients with migraine were enrolled from the neurology outpatient department of the Tri-Service General Hospital (TSGH) between February 2019 and May 2021. Patients enrolled in this study were genetically unrelated. The study protocol was approved by the institutional review board of the Tri-Service General Hospital (TSGHIRB No: 2-108-05-038). All participants provided signed informed consent prior to enrollment. The migraine group was divided into subgroups of migraine with depression and migraine without depression (Figure 1). All migraine groups were then stratified according to migraine subtype and comorbid depression: episodic migraine (EM, EM with depression; EM without depression), chronic migraine (CM, CM with depression; CM without depression), migraine with aura (MA, MA with depression; MA without depression), and migraine without aura (MoA with depression; MoA without depression) (Figure 1).

### 2.2. Migraine and depression assessments

The migraine diagnosis was based on criteria from the third edition of the International Classification of Headache Disorders (ICHD-3) (15). Patients with concurrent primary or secondary headache disorders were excluded from the study. All patients were interviewed by a board-certified neurologist and headache specialist (FCY). Detailed medical histories were obtained and recorded, including aura symptoms, headache duration (years), headache frequency (headache days per month), headache intensity, and family history. All patients were asked to complete several standardized questionnaires, including a demographic information questionnaire, the Migraine Disability Assessment Test (MIDAS) (16), the Hospital Anxiety and Depression Scale (HADS) (17), and the Beck Depression Inventory (BDI) (18). The MIDAS test was a five-item questionnaire that assessed headache-related disabilities in the last 3 months, with a total score ranging from 0 to 90. This test was used to assign disability grading (grades I–IV), with higher disability grades indicating more severe disability (16). The HADS contained seven items related to anxiety and depression, with a maximum subscale score of 21 (17), whereas the total BDI score ranged from 0 to 63 (18). The clinical diagnosis of depression was based on the DSM-5. The exclusion criteria for all participants were lifetime alcohol or substance abuse, intellectual disability, history of major head trauma, and other neurological or neurodegenerative illnesses.

### 2.3. Genotyping and quality control

Peripheral blood from patients with migraine was collected in 5 ml EDTA vacutainers (BD, Plymouth, UK), and genomic DNA was extracted using the QIAamp DSP DNA Mini Kit on a QIAsymphony platform (QIAGEN, Hilden, Germany). DNA quality was assessed using a NanoDrop One spectrophotometer (Thermo Fisher Scientific, Waltham, MA, USA). These DNA samples were applied to the Affymetrix Axiom Genome-Wide TWB 2.0, which contains approximately 752,921 probes for a total of 686,463 single nucleotide polymorphisms (SNPs) (19). Among these SNPs, approximately 446,000 SNPs are associated with the characteristics of background genotypes in Taiwanese, approximately 105,000 SNPs were clinically significant, and others were associated with disease, drug response, and metabolism, which have been determined by Thermo Fisher Scientific for many years. The signal CEL files generated from Axiom TWB 2.0 SNP array were transformed to genotyping data (tped and tfam) using Genotyping Console (Affymetrix).

### 2.4. Statistical analysis

We collected all demographic questionnaires using standard operating procedures for migraine patients. To compare the co-variants between migraine and depression, phenotype association studies were performed using PLINK (20) based on the groups, those with and without depression. The *P*-value and odds ratio (OR) in the phenotype association study were calculated to study the variant relationship using the 1 df chi-square allelic test. Furthermore, all migraine cohorts were subdivided based on four clinical conditions: EM, CM, MA, and MoA. The variants in each group were assessed using a phenotype association study in the Plink association program. In multivariate association analysis, we chose multinomial logit model with 1,000 times bootstraps to build the analysis on genotypes in our findings to migraine frequency, MIDAS, BDI, HADS-anxiety, and HADS-depression scores and corrected it with age, sex, and principal component scores. In addition, we searched 101 variants from previous findings in the Taiwan Biobank (TWB) array. Finally, the suggestive significant variants were retrieved with *P*-values < 1E-05 and were annotated with NCBI based on the RefSeq database (21) using ANNOVAR (22).

## 3. Results

### 3.1. Patient demographic characteristics

Table 1 lists the demographic data of all migraine patients recruited in this study. We divided the patients into those with or without depression and grouped them according to their migraine subtypes: EM, CM, MA, and MoA. There were no significant differences observed in sex, age, migraine duration, body mass index, and education years between the depression and non-depression groups. Migraine frequency, EM/CM, with aura/without aura, MIDAS score, BDI score, HADS-anxiety score, and HADS-depression score presented in Table 1 showed significant differences between subgroups (*P* < 0.05).

**TABLE 1:** Demographics of clinical data.

Section	All migraine	All migraine		*P*-value* (All migraine)
		Non-depression	Depression	
Cohort	966	637	329	–
With aura/without aura	259/707	145/492	114/215	1.06E-04
EM/CM	797/169	556/81	241/88	1.00E-05
Migraine frequency	6.97 ± 7.15	5.95 ± 6.19	8.97 ± 8.38	1.81E-08
Migraine duration, years	26.70 ± 17.82	27.06 ± 17.86	26.01 ± 17.76	3.85E-01
Sex (male/female)	221/729	153/472	68/257	0.25
Age, years	46.77 ± 13.86	47.15 ± 13.90	46.03 ± 13.76	2.39E-01
Body mass index	23.67 ± 4.19	23.64 ± 4.26	23.72 ± 4.05	7.73E-01
Education, years	13.81 ± 3.10	13.87 ± 3.06	13.71 ± 3.17	4.55E-01
MIDAS score	19.11 ± 16.89	16.80 ± 15.53	23.51 ± 18.46	5.43E-08
BDI score	11.93 ± 8.99	6.64 ± 3.62	22.18 ± 7.27	2.25E-131
HADS-anxiety score	7.63 ± 4.15	5.98 ± 3.37	10.83 ± 3.63	3.33E-69
HADS-depression score	6.18 ± 4.14	4.40 ± 3.07	9.64 ± 3.74	2.01E-76

*P-values were calculated using Chi-square test and *t*-test grouped by depression and non-depression. EM, episodic migraine; CM, chronic migraine; MIDAS, Migraine Disability Assessment Scale; BDI, Beck Depression Inventory; HADS, Hospital Anxiety and Depression Scale.

### 3.2. Association of depression in all migraine cohort

**FIGURE 2:**
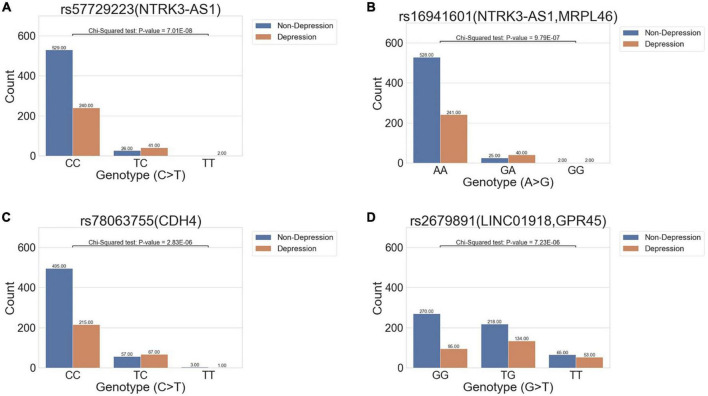
Bar charts show the distribution of variant allele frequency of the variant in all migraine cohort between the non-depression and depression groups. The x-axis denotes the genotype of the variants, and the y-axis indicates the count of genotypes in the two phenotypes. The abundance of each condition in the genotypes is marked above each bar. Four variants **(A)** rs57729223 (*P* = 7.01E-08, OR = 3.60), **(B)** rs16941601 (*P* = 9.79E-07, OR = 3.14), **(C)** rs78063755 (*P* = 2.83E-06, OR = 2.30), and **(D)** rs2679891 (*P* = 7.23E-06, OR = 1.61) were found to be suggestively associated with depression in all migraine cohorts.

All migraine patients were divided into two groups, with or without comorbid depression, for analysis. The GWAS results revealed four suggestive significant variants with *P*-values < 1E-05, all of which had an OR >1, indicating a statistically significant increase in variant allele frequency in the migraine–depression group compared to that without depression. The variants were rs57729223 (*P* = 7.01E-08, OR = 3.60), rs16941601 (*P* = 9.79E-07, OR = 3.14), rs78063755 (*P* = 2.83E-06, OR = 2.30), and rs2679891 (*P* = 7.23E-06, OR = 1.61) (Table 2 and Figure 2).

**TABLE 2:** Association among all migraine patients with depression.

Group	SNP	Position (GRCh38.p12)	EAF	TWB	Gene	Type	Variant change	Variant allele frequency		OR	*P*-value	Adjusted *P*-value
								Non-depression	Depression			
All Migraine cohort	rs57729223	chr15:88271981	0.03	0.04	NTRK3-AS1	Downstream	C > T	2.34%	7.95%	3.60 (2.20, 5.90)	7.01E-08	1.14E-07
	rs16941601	chr15:88281169	0.02	0.04	NTRK3-AS1, MRPL46	Intergenic	A > G	2.61%	7.77%	3.14 (1.94, 5.08)	9.79E-07	1.47E-06
	rs78063755	chr20:61460980	0.12	0.06	CDH4	Intronic	C > T	5.68%	12.19%	2.30 (1.61, 3.30)	2.83E-06	4.10E-06
	rs2679891	chr2:105174855	0.35	0.35	LINC01918, GPR45	Intergenic	G > T	31.46%	42.55%	1.61 (1.31, 1.99)	7.23E-06	1.02E-05

Suggestive significant variants with *P*-values < 1E-5 are listed with allele frequency and OR. EAF, effective allele frequency of the East Asian group in dbSNP; TWB, allele frequency in Taiwan Biobank. The method for adjust *p*-value is genomic-control corrected *p*-values. The Lambda GC in all migraine was 1.03.

### 3.3. Association of depression in the migraine subgroups

#### 3.3.1. EM/CM

**FIGURE 3:**
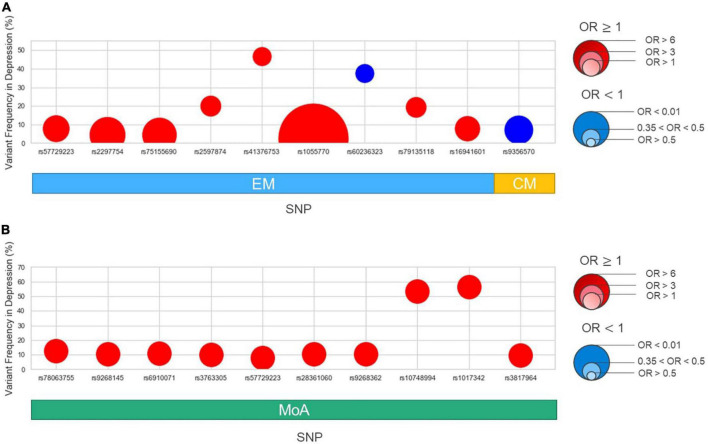
Variant frequency and odds ratio in subgroups: episodic migraine (EM), chronic migraine (CM), aura (MA), and non-aura (MoA). The x-axis shows the variants identified in this study, subgrouping into EM/CM **(A)** and MA/MoA **(B)** as in Table 2. The y-axis shows the variant allele frequency in the depression group. The size of dots is based on the odds ratio. Red in each point denotes OR > 1, while blue denotes OR < 1.

We then performed an association analysis based on migraine subtypes with or without comorbid depression. In the EM/CM and the with or without comorbid depression subgroups (EM-depression, EM-non-depression, CM-depression, CM-non-depression), nine genome-wide suggestive significant (*P* < 1E-05) variants were identified in the EM group (Table 3 and Figure 3A), and two SNPs (rs57729223, rs16941601) were also identified in the migraine cohort (Table 2). One variant was identified in the CM group (Table 3 and Figure 3A), meanwhile, eight variants (all in the EM group) showed OR > 1, and two variants (one in EM, one in CM) had an OR < 1 (Table 3).

**TABLE 3:** Association among episodic migraine (EM), chronic migraine (CM), migraine with aura (MA), and migraine without aura (MoA) with depression.

Group	SNP	Position (GRCh38.p12)	EAF	TWB	Gene	Type	Variant change	Variant allele frequency		OR	*P*-value	Adjusted *P*-value
								Non-depression	Depression			
EM	rs57729223	chr15:88271981	0.025	0.04	NTRK3-AS1	Downstream	C > T	2.44%	8.05%	3.49 (2.04, 5.99)	1.51E-06	1.63E-06
	rs2297754	chr1:147213525	0.029	0.017	CHD1L, FMO5	Intronic	T > C	0.72%	4.39%	6.37 (2.64, 15.37)	2.65E-06	2.65E-06
	rs75155690	chr10:123161005	0.009	0.013	BUB3	Intronic	C > T	0.82%	4.66%	5.87 (2.55, 13.53)	2.79E-06	2.79E-06
	rs2597874	chr4:111615513	0.843	0.85	MIR297, FAM241A	Intergenic	C > A	10.69%	20.00%	2.09 (1.52, 2.86)	3.45E-06	3.72E-06
	rs41376753	chr7:143939387	0.42	0.38	OR2F2, OR2F1	Intergenic	C > T	33.60%	46.59%	1.72 (1.36, 2.18)	5.07E-06	5.45E-06
	rs1055770	chr2:85787657	0.011	0.016	ATOH8	UTR3	A > G	0.10%	2.46%	24.72 (3.15, 193.8)	6.54E-06	7.00E-06
	rs60236323	chr5:140552192	0.59	0.43	SRA1	Intronic deletion	GG >	50.71%	37.5%	0.58 (0.12, 0.46)	7.38E-06	7.90E-06
	rs79135118	chr12:41244499	0.15	0.16	PDZRN4	Intronic	A > G	10.41%	19.27%	2.05 (1.49, 2.83)	7.60E-06	8.14E-06
	rs16941601	chr15:88281169	0.02	0.04	NTRK3-AS1, MRPL46	Intergenic	A > G	2.75%	8.05%	3.1 (1.84, 5.22)	9.09E-06	9.72E-06
CM	rs9356570	chr6:167218800	0.5	0.13	TCP10L2, HPAT5	Intergenic	T > C	24.17%	7.29%	0.25 (0.13, 0.46)	2.92E-06	5.694e-06
MoA	rs78063755	chr20:61460980	0.12	0.06	CDH4	Intronic	C > T	5.06%	12.83%	2.76 (1.8, 4.24)	1.54E-06	1.54E-06
	rs9268145	chr6:32289507	0.089	0.07	TSBP1-AS1	ncRNA_ intronic	T > G	3.91%	10.70%	2.95 (1.83, 4.73)	3.47E-06	3.47E-06
	rs6910071	chr6:32315077	0.084	0.08	TSBP1-AS1	ncRNA_ intronic	A > G	4.02%	10.75%	2.87 (1.79, 4.6)	5.10E-06	5.10E-06
	rs3763305	chr6:32401711	0.077	0.073	TSBP1-AS1	ncRNA_ intronic	G > A	3.68%	10.16%	2.96 (1.82, 4.82)	5.38E-06	5.38E-06
	rs57729223	chr15:88271981	0.03	0.04	NTRK3-AS1	downstream	C > T	2.30%	7.75%	3.57 (1.99, 6.4)	5.75E-06	5.75E-06
	rs28361060	chr6:32336071	0.06	0.08	TSBP1-AS1	ncRNA_ intronic	G > A	4.02%	10.70%	2.86 (1.78, 4.58)	5.80E-06	5.80E-06
	rs9268362	chr6:32365564	0.085	0.08	TSBP1-AS1	ncRNA_ intronic	A > G	4.02%	10.70%	2.86 (1.78, 4.58)	5.80E-06	5.80E-06
	rs10748994	chr10:82477873	0.56	0.41	NRG3	intronic	A > G	39.63%	53.48%	1.75 (1.37, 2.24)	6.44E-06	6.44E-06
	rs1017342	chr8:124701550	0.49	0.47	MTSS1	intronic	C > T	42.73%	56.42%	1.74 (1.36, 2.22)	9.23E-06	9.23E-06
	rs3817964	chr6:32400220	0.085	0.07	TSBP1-AS1	ncRNA_ intronic	T > A	3.36%	9.46%	3 (1.81, 4.99)	9.91E-06	9.91E-06

Suggestive significant variants with *P*-values < 1E-5 are listed with allele frequency and odds ratio (OR). EM, episodic migraine; CM, chronic migraine; MA, migraine with aura; MoA, migraine without aura; EAF, effective allele frequency of the East Asian group in dbSNP; TWB, allele frequency in Taiwan Biobank. The method for adjust *p*-value is genomic-control corrected *p*-values. The Lambda GC of models were 1.01, 1.09, 1.06, 0.99 in EM, CM, MA, and MoA groups, respectively.

#### 3.3.2. MA/MoA

In the MA/MoA with or without comorbid depression subgroups, ten variants were identified in the MoA group with *P* < 1E-05 (Table 3 and Figure 3B); two SNPs, rs57729223 and rs78063755, were also found in all migraine groups (Table 2). No SNPs were identified in the MA group (Table 3). All variants identified in the MoA group had an OR > 1.

### 3.4. Multivariate association study

We performed a multivariate regression analysis of our findings in Table 2 using the following index: migraine frequency, MIDAS, BDI, HADS-anxiety, and HADS-depression scores with age, sex, and principal component analysis scores (Supplementary Table 1) by bootstrapping estimation of multinomial logit models. In all migraine groups, we found that only MIDAS and BDI scores were significantly associated with a variant in the intron region of *CDH4* rs78063755 (*P*-value = 0.03, 0.048; OR = 1.05, 0.98; 95% confidence interval 1.02–1.08, 0.96–0.99, respectively). The other variants that were identified were not associated with the factors mentioned above.

### 3.5. Replication study

We aimed to validate the results of a previous major GWAS on depression with a majority of European participants (23). We searched 101 variants from previous findings, and only six variants were matched in the Taiwan Biobank (TWB) array. We selected these six loci in the TWB2 SNP array and analyzed them in all migraine patients. No SNPs significantly differed between the migraine patients with and without depression in our cohort (Table 4).

**TABLE 4:** Replication of findings in previous depression studies.

SNP	Position (GRCh38.p12)	EAF	TWB	Gene	Type	Variant change	Variant allele frequency		OR	*P*-value	Adjusted *P*-value	Reference
							Non-depression	Depression				
rs1448938	chr11:30871277	0.06	0.28	DCDC1	Intronic	A > G	28.62%	24.56%	0.81 (0.64, 1.02)	0.077	0.082	Howard et al. (23)
rs72710803	chr1:177458882	0.04	0.05	LINC01645, LINC01741	Intergenic	A > C	5.96%	4.06%	0.67 (0.41, 1.09)	0.10	0.11	
rs9592461	chr13:66367660	0.29	0.29	PCDH9	Intronic	A > G	24.55%	26.95%	1.13 (0.9, 1.43)	0.29	0.29	
rs997934	chr10:1753000	0.53	0.54	ADARB2, LINC00700	Intergenic	T > C	46.75%	47.70%	1.04 (0.85, 1.27)	0.71	0.71	
rs301799	chr1:8429242	0.87	0.83	RERE-AS1	ncRNA_ intronic	C > T	16.13%	15.55%	0.96 (0.73, 1.26)	0.76	0.76	
rs2568958	chr1:72299433	0.92	0.92	NEGR1, LINC01360	intergenic	G > A	9.21%	9.54%	1.04 (0.74, 1.47)	0.82	0.83	

The profiles of variants reported in previous studies were found in all migraine cohorts in the depression groups. EAF, effective allele frequency of the East Asian group in dbSNP; TWB, variant allele frequency in Taiwan Biobank. The method for adjust *p*-value is genomic-control corrected *p*-values.

## 4. Discussion

We analyzed 966 patients with migraine and stratified them into groups with or without depression. SNP variants detected in the intron region of *CDH4* (rs78063755), downstream region of *NTRK3-AS1* (rs57729223), the intergenic region between *NTRK3-AS1* and *MRPL46* (rs16941601), and the intergenic region between *LINC01918* and *GPR45* (rs2679891) were found to be suggestively associated with depression in the Han Chinese population in Taiwan. However, the distance between rs57729223 and rs16941601 was close, and the *r2* measure of linkage disequilibrium (LD) of rs57729223 and rs16941601 was around 0.94 using LDlink (24), indicating that the two SNPs have non-random association and that rs57729223 may be the only one with significance.

Cadherin 4 (*CDH4*) is located on chromosome 20q13.33 and encodes CDH4, a calcium-dependent cell-cell adhesion glycoprotein. *CDH4* is mainly expressed in the brain but also in other tissues, including the spleen, ovary, testis, adrenal gland, and thyroid gland (25). *CDH4* plays a key role in brain segmentation and neuronal outgrowth (26). A GWAS of brain aging on magnetic resonance imaging data showed that the SNP variant in *CDH4* is significantly related to reduced total cerebral brain volume, potentially caused by impairment of the neural tract and synaptic development (27). Another GWAS of depression phenotypes in the UK Biobank also identified SNP variants in *CDH4* as related variants (28). Although *CDH4* polymorphism has been reported as a susceptible locus with respect to migraine (29), the associated mechanism remains unclear. Our study showed that the *CDH4* intronic polymorphism rs78063755 is a candidate for further studies on migraine with depression in the Han Chinese population, possibly due to its impact on brain segmentation, neuronal growth, and synaptic development.

*NTRK3-AS1* is an RNA gene located on chromosome 15q25.3 that is expressed in the testis (25). According to the GWAS catalog, *NTRK3-AS1* polymorphisms are associated with the levels of growth-regulated protein alpha and 3-hydroxypropyl mercapturic acid in smokers (30). However, to the best of our knowledge, no previous study has reported the association between *NTRK3-AS1* polymorphism and migraine or depression. Our analysis also identified another variant, rs57729223, in the downstream region of *NTRK3-AS1*, and two intergenic variants, rs16941601 and rs2679891, which have not previously been reported as associated with migraine and depression. As discussed earlier, because the distance between rs57729223 and rs16941601 was close and the two SNPs are not independent, rs57729223 is considered the one with significance. In addition to all migraine groups, rs57729223 was genetically suggestively associated with comorbid depression in the EM and MoA subgroups. However, the mechanism is unclear and warrants further investigation, particularly in the Han Chinese population.

To investigate whether different SNPs are associated with depression in different migraine types, we analyzed SNP differences between patients with or without depression in different subgroups. Eight SNPs in the EM group and ten in the MoA group were suggestively associated with comorbid depression (OR > 1). One SNP each in EM and CM had OR < 1. In the EM group, we identified nine SNPs suggestively associated with depression (eight with OR > 1; one with OR < 1). Two variants with OR > 1 (rs57729223 and rs16941601) also demonstrated an association with depression in all migraine groups, since rs57729223 and rs16941601 are not independent, the association may be only in rs57729223. Six other SNP variants were suggestively associated with depression in the EM group, including the intronic regions of *CHD2L*, *GMO5*: rs2297754, intronic region of *BUB3*: rs75155690, intronic region of *PDZRN4*: rs79135118, 3’UTR region of *ATOH8*: rs1055770, the intergenic region between *MIR197* and *FAM241A*: rs5897874, and the intergenic region between *OR2F2* and *OR2F1*: rs41376753.

*BUB3* is located on chromosome 10q26.13 and encodes BUB3, a mitotic checkpoint protein belonging to the Bub protein family that plays an important role in the spindle assembly checkpoint (31). Abnormal BUB3 expression causes defective mitotic function, impaired spindle gate function, and chromosome instability, leading to aneuploidy and possibly tumorigenesis (31). The GWAS catalog documented its association with exacerbations in children despite using long-acting beta 2-agonists and plasma metabolite kynurenic acid measurement (30). However, the association between *BUB3* SNP and the pathophysiology of migraine or depression remains unclear and warrants further analysis.

PDZ domain-containing ring finger 4 (*PDZRN4*) is located on chromosome 12q12 and encodes the PDZ domain-containing ring finger 4 (PDZRN4), which belongs to the LNX family and acts as a suppressor in multiple cancers (32, 33) and in association with multiple sclerosis in a GWAS (34). However, to date, no study has discussed its relationship with migraine or depression, and hence may serve as a candidate for further research.

*ATOH8* is located on chromosome 2p11.2 and encodes protein ATOH8, which belongs to a family of transcriptional regulators: basic helix-loop-helix proteins (35). ATOH8 is involved in embryonic development, disease initiation, and disease progression (35). The GWAS catalog reports that SNPs in the *ATOH8* are suggestively associated with optic disk size, abdominal aortic aneurysm, total blood homocysteine level, total cerebrospinal fluid paired helical filament-tau level, and metastasis in low/stable colorectal cancer (30). Meanwhile, the association of ATOH8 with depression or migraine has not been reported, and further studies are needed to delineate the possible mechanisms.

One SNP variant, the intronic deletion in steroid receptor RNA activator 1 (*SRA1*) gene (rs60236323), had an OR < 1 with a variant allele frequency of 50.7% in non-depression EM patients and 37.5% in depression EM patients. The *SRA1* gene is located on chromosome 5q31.3 and has a bi-functional role as both protein and non-coding RNAs (36). Moreover, it is involved in the development of multiple cancers (37, 38) and cardiomyopathy (39). However, its association with migraines and depression remains unclear. Whether this SNP variant has a possible protective role in Han Chinese patients with EM requires further investigation with larger samples.

In the MoA group, we identified ten variants suggestively associated with depression (OR > 1). Two of which (rs78063755 in *CDH4* and rs57729223 in *NTRK3-AS1*) were also found to be associated with depression in the migraine group. Six ncRNA intronic variants (rs9268145, rs6910071, rs3763305, rs28361060, rs9268362, and rs3817964) were located in the same gene *TSBP1-AS1*, and two intronic variants in *NRG3* (rs10748994) and *MTSS1* (rs1017342).

*TSBP1-AS1*, located on chromosome 6q21.32, is an RNA gene affiliated with the lncRNA class (30). The function of *TSBP1-AS1* remains to be identified. Nevertheless, it is highly expressed in immune system cells (40) and genetically associated with several immune-related diseases, hepatitis, and dermatologic disorders, according to the GWAS catalog (30). Interestingly, *TSBP1-AS1* is located in the major histocompatibility complex (MHC) region (41). Previous studies have reported that the clinical features of migraine may be influenced by SNPs located in the MHC region (42, 43). Huang et al. (43) showed that some alleles of *HLA* class I were associated with migraine and increased risks of chronic migraine and medication overuse. However, whether migraine is genetically related to immune disorders warrants further investigation. A previous GWAS identified a *TSBP1-AS1* variant (rs140002913) in association with migraine disorder (44). In our study, we identified six *TSBP1-AS1* variants in association with MoA and comorbid depression with variant allele frequencies 2.86–3 times higher than those without comorbid depression. Further studies are needed to elucidate the role and pathophysiology of *TSBP1-AS1* in migraine and depression.

*NRG3* is located on chromosome 10q23.1 and encodes neuregulin 3, a protein belonging to the neuregulin family that plays important roles in neuronal development, differentiation, proliferation, and plasticity (45, 46). According to the GWAS catalog, *NRG3* polymorphisms are genetically associated with various diseases, including cancer, insulin resistance, and tau protein formation (30). However, the association between migraine and depression has not been reported. We observed that the frequency of the variant allele (rs10748994) was higher in MoA patients with comorbid depression. The underlying mechanism remains unknown, and *NRG3* may serve as a suitable candidate for further studies.

*MTSS1* is located on chromosome 8q24.13 and encodes metastasis suppressor 1, a protein involved in cell morphology, motility, development, and metastasis (47). According to the GWAS catalog, its SNPs were reported to be genetically associated with multiple traits, including cardiovascular disease, tau protein formation, and cognitive function (30). No association between *MTSS1* SNPs and migraine or depression has been reported. We identified a *MTSS1* variant (rs1017432) that may be genetically associated with MoA and comorbid depression. Further studies are needed to validate our findings and explore the underlying mechanisms.

A multivariate association study demonstrated that the SNP variant in the intron region of *CDH4* rs78063755 was associated with MIDAS and BDI scores. Although the association between migraine and the variant rs78063755 has not been widely discussed, it was suggestively associated with multiple migraine-related indices and may serve as a candidate for further studies. We employed Affymetrix Axiom Genome-Wide TWB 2.0, which covers a representative sample of the gene pool in Taiwan and contains approximately 752,921 probes for a total of 686,463 SNPs in this study, whereas only 6 out of 101 SNPs reported in European cohorts (23) were found in the TWB array and none of the SNPs were significantly related to depression and migraine comorbidity in our cohort. This might have been due to all the participants in our study belonging to the Han Chinese population, whereas the previous study focused primarily on a European population.

Our study has several strengths. For instance, the strict diagnostic criteria and designed standard operation protocol allowed us to record detailed patient data, including history and medical evaluation, correct diagnosis made by a headache expert, and evaluations using validated questionnaires. Further statistical analysis, including stratified analysis of migraine subgroups and multivariate regression analysis, allowed us to investigate the genetic association between migraine and comorbid depression. Moreover, we employed Affymetrix’s Axiom Genome-wide TWB 2.0 array, which covers a representative sample of the gene pool in Taiwan (19), and identified several SNPs suggestively associated with migraine or depression. Our study had some limitations. We did not perform genotype imputation prior to performing GWAS because using data from European populations may not necessarily increase the number of matched SNPs and the results obtained may not be applicable in the Asian population. However, further exploration of this study with genotype imputation will be performed when more cross-population whole exome sequencing data are available. We did not perform replication on all the SNPs found in the European population due to limited SNPs in the TWB 2.0 array. Our cohort sample size was relatively modest, and the sub-groups results could not meet a traditionally accepted *P*-value threshold of <5E-08 in GWAS. Hopefully, our exploratory genetic association results may shed light on the genetic links for depression and migraine comorbidity; however, further validation is warranted.

## 5. Conclusion

This study showed that migraine patients with SNP variants in the intron region of *CDH4*: rs78063755, downstream region of *NTRK3-AS1*: rs57729223, and the intergenic region between *LINC01918* and *GPR45*: rs2679891 are suggestively associated with depression in the Han Chinese population in Taiwan. We also identified several SNPs suggestively associated with depression in migraine subgroups (EM, CM, and MoA). These results provide insights into the possible genetic basis of both overall migraine, and its subtypes, and comorbid depression in the Han Chinese population.

## Data availability statement

The datasets presented in this study can be found in online repositories. The names of the repository/repositories and accession number(s) can be found below: European Variation Archive, PRJEB57851.

## Author contributions

M-CT and F-CY: conceptualization and writing—original draft. F-CY, C-LT, C-SL, Y-KL, C-KT, G-YL, YL, and K-SH: data curation. F-CY, M-CT, C-KT, YL, and K-SH: formal analysis. F-CY: methodology and writing—review and editing. All authors have read and approved the final version of the manuscript.
