# Supplementary material for: Identification of genetic risk loci for depression and migraine comorbidity in Han Chinese residing in Taiwan
Source: Front Psychiatry. 2023 Jan 10;13:1067503. doi: 10.3389/fpsyt.2022.1067503 (PMC9871634; doi:10.3389/fpsyt.2022.1067503)
Supplement: Supplementary file 1 [file Table_1.docx]

| **Variant** | **Factors** | **p-value** | **OR** | **Confidence Interval** |
| --- | --- | --- | --- | --- |
| rs78063755 | BDI score | 0.028 | 1.048 | 1.019 -1.078 |
| rs78063755 | MIDAS score | 0.048 | 0.979 | 0.964-0.993 |

**Supplementary Table 1.** Multivariate association study results demonstrate variants associated with BDI score and MIDAS.
