# Supplementary figures and images for: Identification of genetic risk loci for depression and migraine comorbidity in Han Chinese residing in Taiwan
Source: Front Psychiatry. 2023 Jan 10;13:1067503. doi: 10.3389/fpsyt.2022.1067503 (PMC9871634; doi:10.3389/fpsyt.2022.1067503)

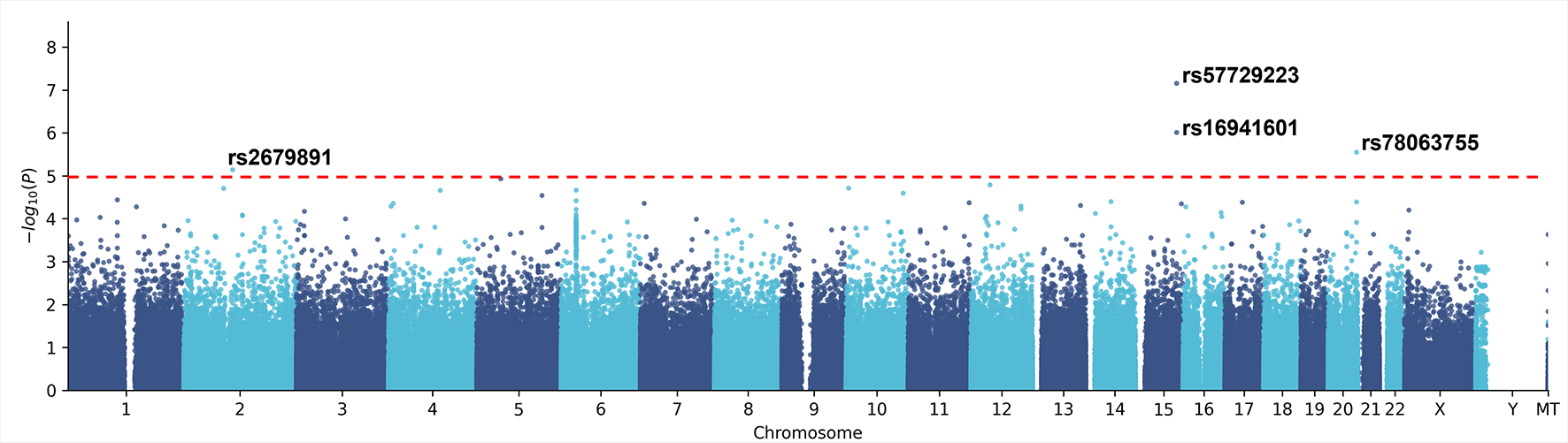

Supplement: Supplementary file 2 [file Image_1.TIF]
